# Bednar Tumor: A Rare Variant of Dermatofibrosarcoma Protuberans

**DOI:** 10.7759/cureus.102955

**Published:** 2026-02-04

**Authors:** Michelle Alcocer Salas, Diana L Vazquez-Cantu, Bryan Salvador Sol Tlapalamatl, Mercedes Hernández Torres, José Alfredo Soto-Ortiz

**Affiliations:** 1 Dermatology, Instituto Dermatológico de Jalisco "Dr. José Barba Rubio", Zapopan, MEX; 2 Dermatopathology, Instituto Dermatológico de Jalisco "Dr. José Barba Rubio", Zapopan, MEX; 3 Dermatology, University of Guadalajara, Guadalajara, MEX

**Keywords:** bednar tumor, cutaneous sarcoma, dermatofibrosarcoma protuberans, immunohistochemistry, pigmented

## Abstract

Dermatofibrosarcoma protuberans (DFSP) is a rare dermal sarcoma of intermediate malignancy characterized by locally aggressive behavior and a low metastatic potential. The classic form typically presents as a slow-growing protuberant tumor involving the trunk or proximal extremities; however, several uncommon variants have been described. We report the case of a 32-year-old woman who presented with a long-standing pigmented lesion on the shoulder that had been present since childhood and showed progressive changes over the preceding six months. Clinically, the lesion evolved into an oval-shaped neoplasm with erythematous and brown pigmentation, a smooth surface, and an infiltrative consistency. Based on the clinical features, a diagnosis of DFSP was suspected, with particular consideration given to its rare pigmented variant, known as Bednar tumor. Histopathological examination and immunohistochemical analysis were performed to confirm the diagnosis and guide management. Although Bednar tumor exhibits distinctive histologic features due to the presence of melanin-producing dendritic cells, its biological behavior and prognosis are similar to those of conventional DFSP. Notably, only three cases of DFSP have been diagnosed over a 13-year period at a tertiary dermatology referral center in western Mexico, underscoring the rarity of this entity and the importance of clinical awareness for timely diagnosis and appropriate treatment.

## Introduction

Dermatofibrosarcoma protuberans (DFSP) is an uncommon dermal sarcoma of intermediate malignancy. It predominantly affects young to middle-aged adults and represents approximately 0.1% of all cancers and 1% of soft tissue sarcomas [1,2]. Most cases are classified as low-grade tumors characterized by slow growth and a marked tendency for local recurrence. In contrast, a smaller subset demonstrates high-grade sarcomatous transformation, which is associated with more aggressive behavior and a low but documented risk of metastasis [3,4]. Clinically, DFSP most often presents as a slowly enlarging, protuberant tumor involving the trunk or proximal extremities.

Several uncommon variants have been described. The pigmented variant, known as Bednar tumor, may pose a diagnostic challenge due to its clinical and histologic resemblance to melanocytic lesions such as melanoma or blue nevus, which can lead to misdiagnosis and delayed definitive treatment [5].

## Case presentation

**Figure 1:**
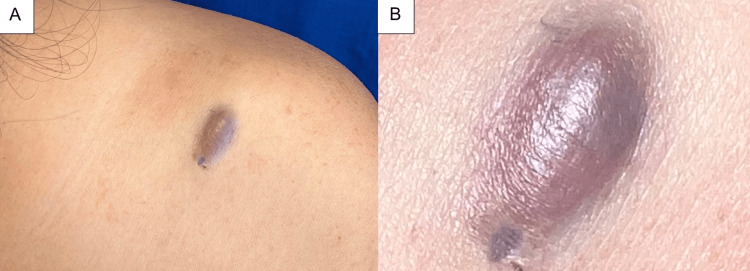
Clinical presentation (A) Oval erythematous-violaceous neoformation with a smooth surface on the posterior aspect of the left shoulder. (B) Close-up view of the lesion

A 32-year-old woman with no significant medical history was referred to the Instituto Dermatológico de Jalisco, a tertiary dermatology referral center in western Mexico, because of progressive growth and pain over six months in a pigmented lesion that had been present since childhood. The lesion was located on the posterior aspect of the left shoulder and presented as an oval erythematous neoformation measuring 1.5 × 0.5 × 0.3 cm, with a focal brown pigmentation, a smooth surface, an indurated consistency, and well-defined borders (Figure 1A, 1B).

**Figure 2:**
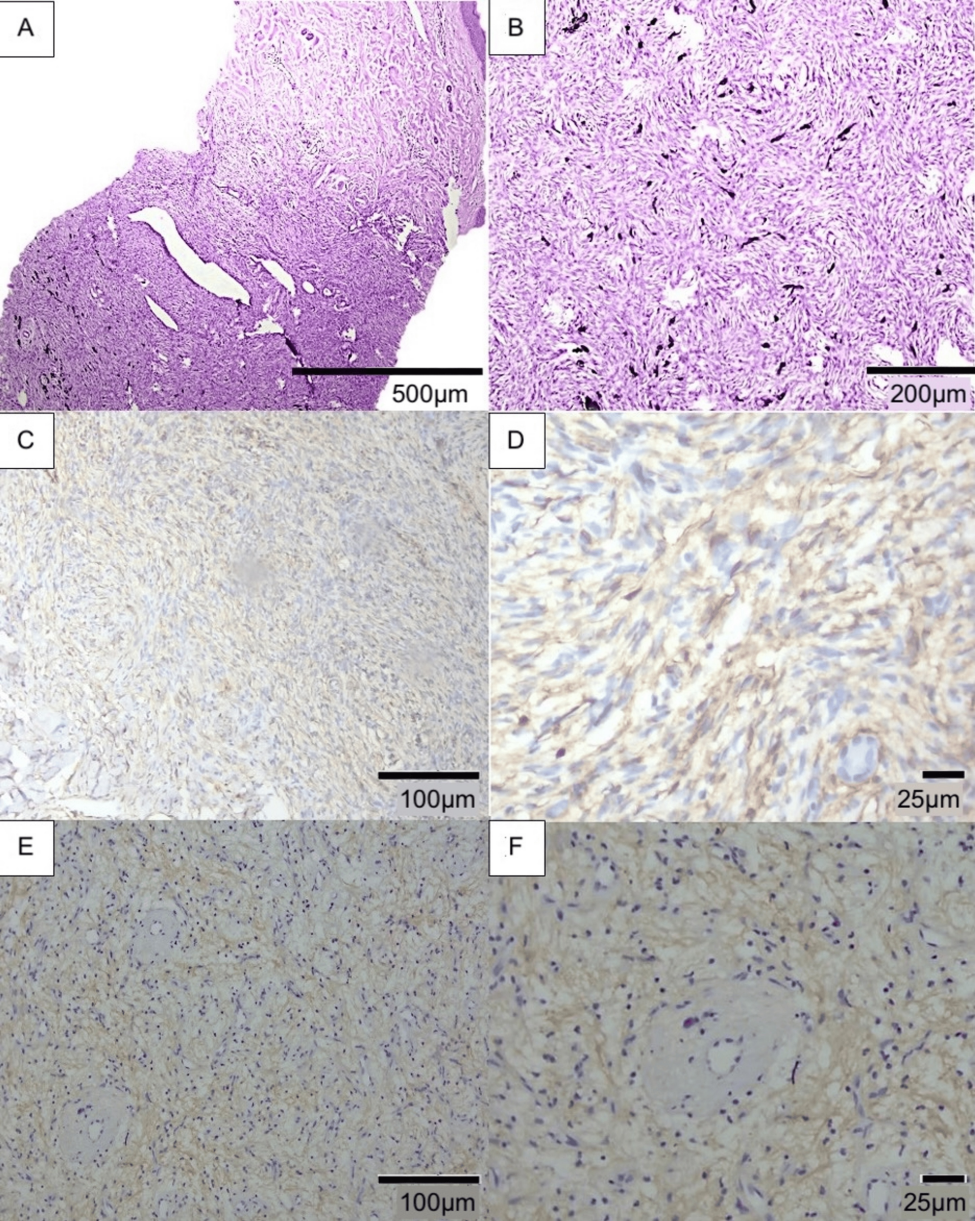
Histopathological and immunohistochemical findings (A) Flattened epidermis with a dermal spindle cell proliferation extending into the subcutaneous tissue (H&E: ×40). (B) Higher-magnification view showing spindle cells arranged in a storiform pattern with interspersed pigment (H&E: ×200). (C and D) Diffuse immunoreactivity for CD34 within tumor cells (C, ×100; D, ×400). (E and F) Absence of factor XIIIa immunoreactivity (E, ×100; F, ×400) CD34: cluster of differentiation 34

A punch biopsy was performed and revealed histopathological findings consistent with the pigmented variant of DFSP, known as Bednar tumor. Immunohistochemical analysis demonstrated diffuse positivity for cluster of differentiation 34 (CD34) and negative staining for factor XIIIa (Figure 2A-2F). No regional lymphadenopathy was identified on physical examination. According to the American Joint Committee on Cancer's Cancer Staging Manual, Eighth Edition, the lesion was classified as clinical stage IA, corresponding to a localized tumor without invasion beyond the fascia [6].

**Figure 3:**
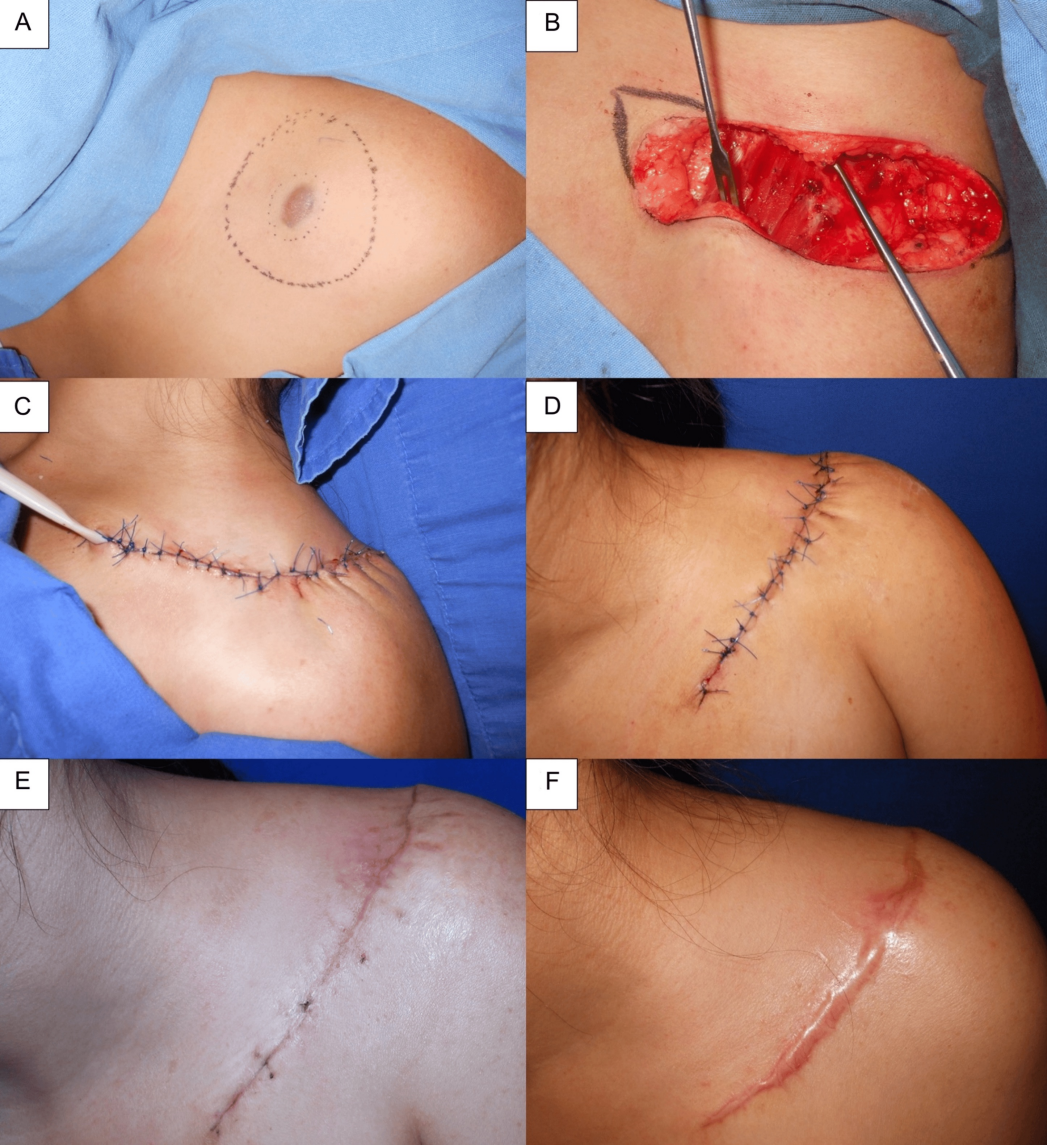
Surgical management and follow-up (A) Preoperative tumor delineation with a 2.5 cm surgical margin. (B) Tumor resection down to the fascia, followed by primary layered closure. (C) Immediate postoperative appearance. (D and E) Follow-up images at five days, one month, and six months, demonstrating the development of a keloid scar

The patient underwent a wide local excision with 2.5 cm surgical margins, including fasciectomy, followed by primary layered closure (Figure 3A-3C). At six months of follow-up, there was no evidence of tumor recurrence. The postoperative course was complicated by the development of a keloid scar, which has been managed with intralesional triamcinolone acetonide injections, with clinical improvement (Figure 3D-3F).

## Discussion

DFSP is a soft tissue sarcoma that primarily affects young adults, most commonly during the third decade of life [1]. According to Liszewski et al., in a cohort of 5,139 patients with DFSP, the trunk was the most frequently involved site (47.2%), followed by the lower extremities (20.0%), upper extremities (19.3%), head and neck (11.1%), and genital region (1.3%) [7]. Most cases corresponded to the classic form, while only 2.5% were classified as the pigmented variant, Bednar tumor. This variant has been reported more frequently in non-Hispanic White and African American populations, contrasting with earlier studies suggesting a higher incidence in Black individuals [8]. In our patient, who was of Hispanic descent, the lesion was located on the trunk.

From a molecular standpoint, more than 90% of cases harbor a characteristic chromosomal translocation t(17;22)(q22;q13), resulting in the fusion of the platelet-derived growth factor beta (*PDGFB*) gene with the collagen type I alpha 1 (*COL1A1*) gene, which drives tumor proliferation [3]. Bednar tumor is believed to have a neuroectodermal origin and is characterized by the presence of dendritic, melanin-containing cells interspersed among the typical spindle-shaped cells. Epidemiological data suggest that factors beyond race and skin phototype may contribute to pigmentation in this variant [9].

Clinically, DFSP usually presents as a slowly growing, asymptomatic, skin-colored or erythematous plaque that may evolve into nodular or protuberant lesions, occasionally accompanied by ulceration or bleeding [10]. Non-protuberant forms are less common and include morphea-like, atrophoderma-like, and angioma-like variants, classified according to their clinical resemblance to these conditions [11].

Diagnosis relies on clinicopathological correlation and varies according to disease stage. In the plaque phase, spindle cells are typically arranged in parallel fascicles with low mitotic activity and may infiltrate the subcutaneous tissue in a tentacle-like pattern. In the nodular phase, increased mitotic activity and nuclear atypia are observed, with tumor cells arranged in a honeycomb pattern. In the pigmented variant, melanocyte-containing dendritic cells with melanocytic or schwannian differentiation are present within the tumor [11].

Over the past 13 years (2011-2024), two additional patients were diagnosed histopathologically at the Instituto Dermatológico de Jalisco. Among the three total cases, two occurred in female patients and one in a male patient, with a median age at diagnosis of 32 years and a median lesion evolution time of 18 months. Two lesions (66%) were located on the trunk, specifically the shoulder, while one involved the face. All lesions presented as erythematous-violaceous neoplasms of variable size (Table 1).

**Table 1:** Clinical features of patients with histopathologically confirmed Bednar tumor at the Instituto Dermatológico de Jalisco (2011-2024)

Number	Patient	Age (years)	Sex	Evolution	Location	Tumor morphology	Tumor size	Clinical diagnosis	Treatment
1	Case 1	26	Female	12 months	Shoulder	Erythematous oval neoplasm	4 × 2 × 1 cm	Aneurysmal fibrous histiocytoma	Excision
2	Case 2	43	Male	18 months	Face	Erythematous oval neoplasm	7 × 7 × 1 mm	Cutaneous adnexal tumor	Excision
3	Index patient	32	Female	Since childhood	Shoulder	Erythematous oval neoplasm	15 × 5 × 3 mm	Vascular malformation	Excision

Histopathological examination is essential for diagnosis; however, immunohistochemical analysis is recommended to differentiate DFSP from other mesenchymal tumors. Fibrosarcomatous transformation represents a more aggressive variant and is characterized by the loss of the storiform pattern, a herringbone or spiked appearance, and reduced CD34 expression. In contrast, conventional cases typically show strong positivity for CD34, hyaluronate, and vimentin [11,12]. In diagnostically challenging cases, molecular techniques may be used to confirm the presence of the characteristic *COL1A1*-*PDGFB* gene fusion [12].

Surgical management remains the cornerstone of treatment and is tailored to the tumor extent. For large, recurrent, or anatomically complex lesions, Mohs micrographic surgery or peripheral and deep margin assessment is preferred to ensure complete margin control. In localized tumors or when these techniques are unavailable, a wide local excision is recommended. The National Comprehensive Cancer Network advises surgical margins of 2-4 cm, including the resection of the underlying muscle fascia when tumors exceed 2 cm in size [13].

In the present case, a wide local excision with 2.5 cm margins and fasciectomy was performed. Histopathological evaluation revealed close surgical margins, prompting the addition of adjuvant radiotherapy. In cases with positive margins, re-excision is recommended whenever feasible; alternatively, radiotherapy or systemic therapies may be considered [13]. For locally advanced, recurrent, or metastatic disease, targeted therapy with tyrosine kinase inhibitors has demonstrated response rates of approximately 50%-60% [13]. Overall, surgical margin status remains the most important prognostic factor, and patients generally have a favorable prognosis.

## Conclusions

This case highlights a less common clinical variant of DFSP known as Bednar tumor. The recognition of the diverse clinical and histopathological presentations of DFSP is essential for timely and accurate diagnosis, particularly given its resemblance to other entities such as fibrous histiocytoma, blue nevus, and malignant melanoma. The diagnosis of Bednar tumor requires a comprehensive immunohistochemical panel, including CD34 and factor XIIIa, to differentiate it from other spindle cell neoplasms. The identification of only three cases over a 13-year period at a tertiary dermatology referral center in western Mexico underscores the rarity of this entity and the importance of maintaining a high index of suspicion.

## References

[1] Bowne WB, Antonescu CR, Leung DH (2000). Dermatofibrosarcoma protuberans: a clinicopathologic analysis of patients treated and followed at a single institution. Cancer.

[2] Molinari L, Luna AM, Ferrario D, Galimberti G, Galimberti R (2015). Dermatofibrosarcoma protuberans: a review (Article in Spanish). Dermat Cosmet Méd Quir.

[3] Salgado R, Llombart B, M Pujol R (2011). Molecular diagnosis of dermatofibrosarcoma protuberans: a comparison between reverse transcriptase-polymerase chain reaction and fluorescence in situ hybridization methodologies. Genes Chromosomes Cancer.

[4] Rutgers EJ, Kroon BB, Albus-Lutter CE, Gortzak E (1992). Dermatofibrosarcoma protuberans: treatment and prognosis. Eur J Surg Oncol.

[5] Hali F, Idrissi OK, Jadib I, Bachkira M, Hadad O (2023). Unusual presentation of dermatofibrosarcoma protuberans. Int J Clin Med Case Rep.

[6] (2017). AJCC Cancer Staging Manual. AJCC Cancer Staging Manual. 8th ed. New York: Springer.

[7] Liszewski W, Blanchette D, Cunningham AM, Miller DD (2016). Epidemiology of Bednar tumors in the United States. J Am Acad Dermatol.

[8] Yagi Y, Ueda K, Maruyama S, Noborio R (2004). Bednar tumor: a report of two cases. J Dermatol.

[9] Dupree WB, Langloss JM, Weiss SW (1985). Pigmented dermatofibrosarcoma protuberans (Bednar tumor). A pathologic, ultrastructural, and immunohistochemical study. Am J Surg Pathol.

[10] Taylor HB, Helwig EB (1962). Dermatofibrosarcoma protuberans. A study of 115 cases. Cancer.

[11] Allen A, Ahn C, Sangüeza OP (2019). Dermatofibrosarcoma protuberans. Dermatol Clin.

[12] Hao X, Billings SD, Wu F, Stultz TW, Procop GW, Mirkin G, Vidimos AT (2020). Dermatofibrosarcoma protuberans: update on the diagnosis and treatment. J Clin Med.

[13] Vitiello GA, Lee AY, Berman RS (2022). Dermatofibrosarcoma protuberans: what is this?. Surg Clin North Am.

